# Computing the orientational-average of diffusion-weighted MRI signals: a comparison of different techniques

**DOI:** 10.1038/s41598-021-93558-1

**Published:** 2021-07-12

**Authors:** Maryam Afzali, Hans Knutsson, Evren Özarslan, Derek K. Jones

**Affiliations:** 1 Cardiff University Brain Research Imaging Centre (CUBRIC), School of Psychology, Cardiff University, Cardiff, CF24 4HQ UK; 2 Leeds Institute of Cardiovascular and Metabolic Medicine, University of Leeds, Leeds, LS2 9JT UK; 3 Department of Biomedical Engineering, Linköping University, 581 83 Linköping, Sweden; 4 Center for Medical Image Science and Visualization, Linköping University, 581 83 Linköping, Sweden

**Keywords:** Computational science, Biomedical engineering

## Abstract

Numerous applications in diffusion MRI involve computing the orientationally-averaged diffusion-weighted signal. Most approaches implicitly assume, for a given b-value, that the gradient sampling vectors are uniformly distributed on a sphere (or ‘shell’), computing the orientationally-averaged signal through simple arithmetic averaging. One challenge with this approach is that not all acquisition schemes have gradient sampling vectors distributed over perfect spheres. To ameliorate this challenge, alternative averaging methods include: weighted signal averaging; spherical harmonic representation of the signal in each shell; and using Mean Apparent Propagator MRI (MAP-MRI) to derive a three-dimensional signal representation and estimate its ‘isotropic part’. Here, these different methods are simulated and compared under different signal-to-noise (SNR) realizations. With sufficiently dense sampling points (61 orientations per shell), and isotropically-distributed sampling vectors, all averaging methods give comparable results, (MAP-MRI-based estimates give slightly higher accuracy, albeit with slightly elevated bias as b-value increases). As the SNR and number of data points per shell are reduced, MAP-MRI-based approaches give significantly higher accuracy compared with the other methods. We also apply these approaches to in vivo data where the results are broadly consistent with our simulations. A statistical analysis of the simulated data shows that the orientationally-averaged signals at each b-value are largely Gaussian distributed.

## Introduction

Diffusion MRI is a non-invasive technique that is sensitive to differences in tissue microstructure, which comprises a combination of micro-environments with potentially different orientational characteristics. Inherent to MRI is averaging of the magnetization across the voxel. However, some orientational features (macroscopic or ensemble anisotropy) typically survive such averaging, facilitating applications like fiber-orientation mapping. However, as far as studies aiming to understand the underlying microstructure are concerned, the presence of such macroscopic anisotropy may introduce complications in the interpretation of the signal. In analogy with solid-state NMR applications^1^, considering the “powdered” structure of the specimen that features replicas of each and every microscopic domain oriented along all possible directions could reveal the desired microstructural properties of the medium more clearly^2,3^.

**Figure 1:**
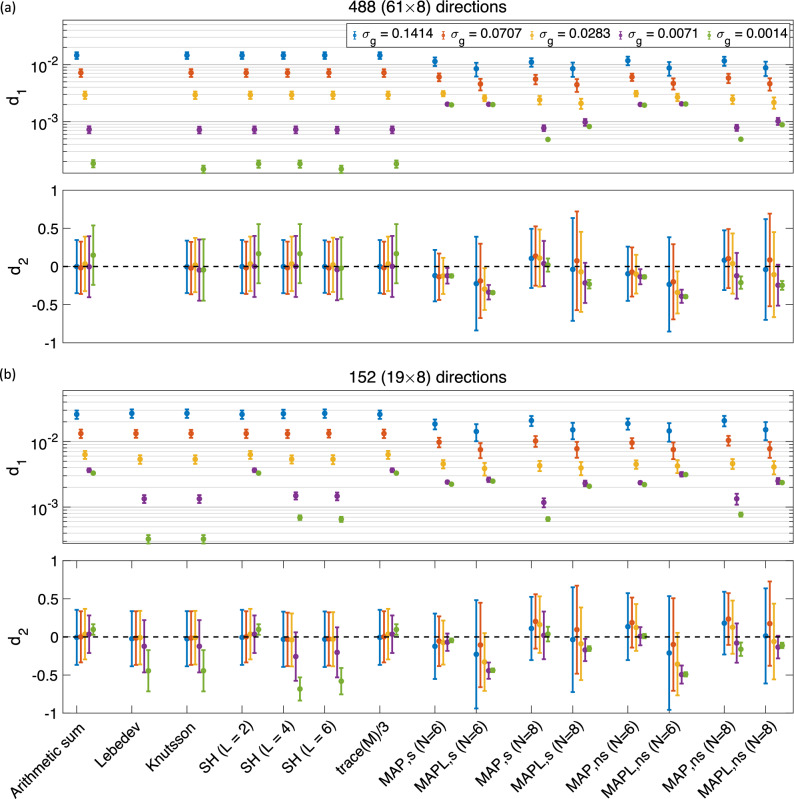
The mean and std of the $d_1$ and $d_2$ measures (illustrated as, respectively, a dot and an error bar) for different methods and different sampling schemes, in the presence of Gaussian noise (**a**) 488 ($61\times 8$)^21^, and (**b**) 152^21^ ($19\times 8$)^31^ directions (the y-axis in $d_1$ is scaled logarithmically). **Arithmetic sum:** simple arithmetic averaging; **Lebedev:** weighted averaging by^31^; **Knutsson:** weighted averaging by^43^; **SH:** Spherical harmonic method for powder averaging by^3^ $L = 2, \; 4, \; 6$, shows the order in spherical harmonic representation; **trace(M)/3:** powder average signal from Eq. (10); **MAP:** direction-averaged signal using MAP-MRI^44^ for $N_{\max} = 6$ and 8; and **MAPL:** direction-averaged signal using MAP-MRI with Laplacian regularization^51^. The ‘s’ and ‘ns’ correspond to the shelled and non-shelled point sets, respectively. Different colors (blue, red, yellow, purple, green) show the results in different noise levels ($\sigma _g = 0.1414,\, 0.0707,\, 0.0283,\, 0.0071,\, 0.0014$).

**Figure 2:**
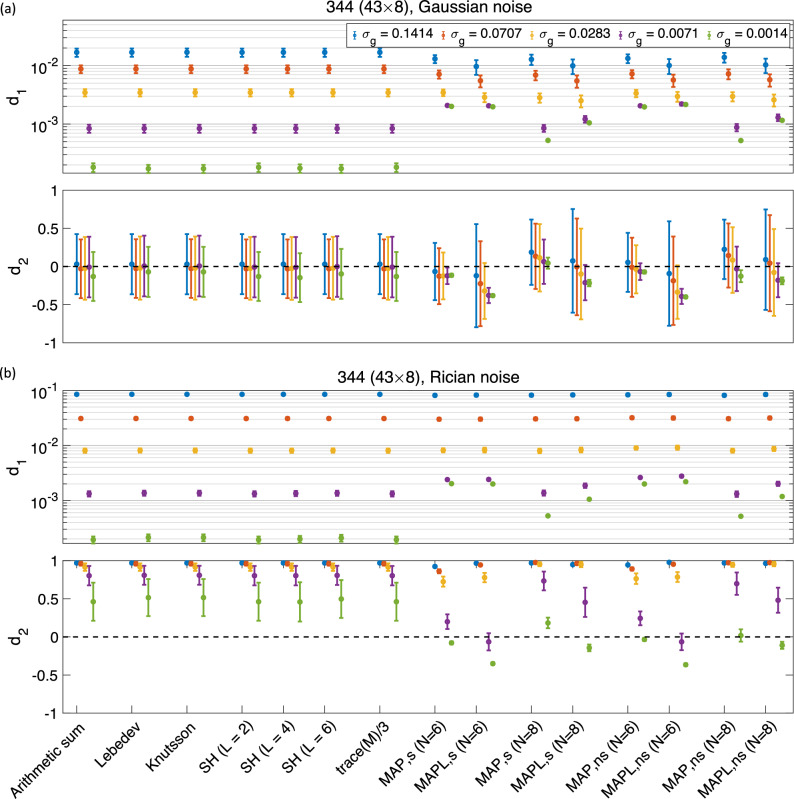
The mean and std of the $d_1$ and $d_2$ measures using different methods for 344 samples, both shelled ($43\times 8$)^31^ and non-shelled^21^ point sets in the presence of (**a**) Gaussian and (**b**) Rician noise (the y-axis in $d_1$ is scaled logarithmically). The ‘s’ and ‘ns’ correspond to the shelled and non-shelled point sets, respectively. Different colors (blue, red, yellow, purple, green) show the results in different noise levels ($\sigma _g = 0.1414, \,0.0707, \,0.0283, \,0.0071,\, 0.0014$).

**Figure 3:**
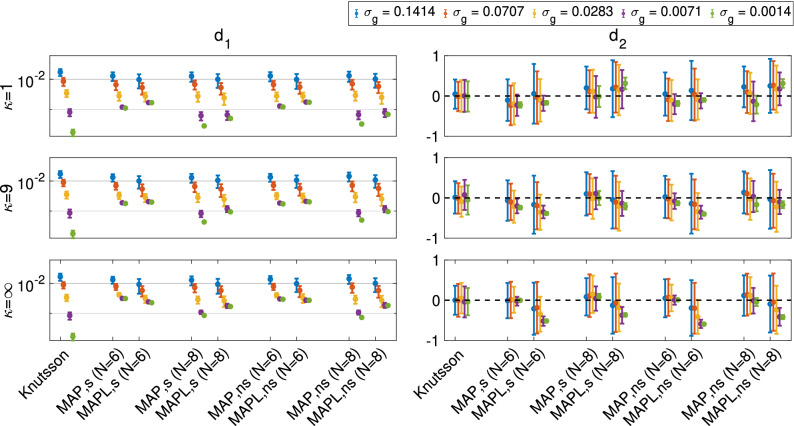
The estimated $d_1$ and $d_2$ for three different $\kappa$ values, 344 ($43 \times 8$) point sets in the presence of five different Gaussian noise levels (the y-axis in $d_1$ is scaled logarithmically). Note that when $\kappa = \infty$ there is no dispersion; decreasing $\kappa$ increases the dispersion.

**Figure 4:**
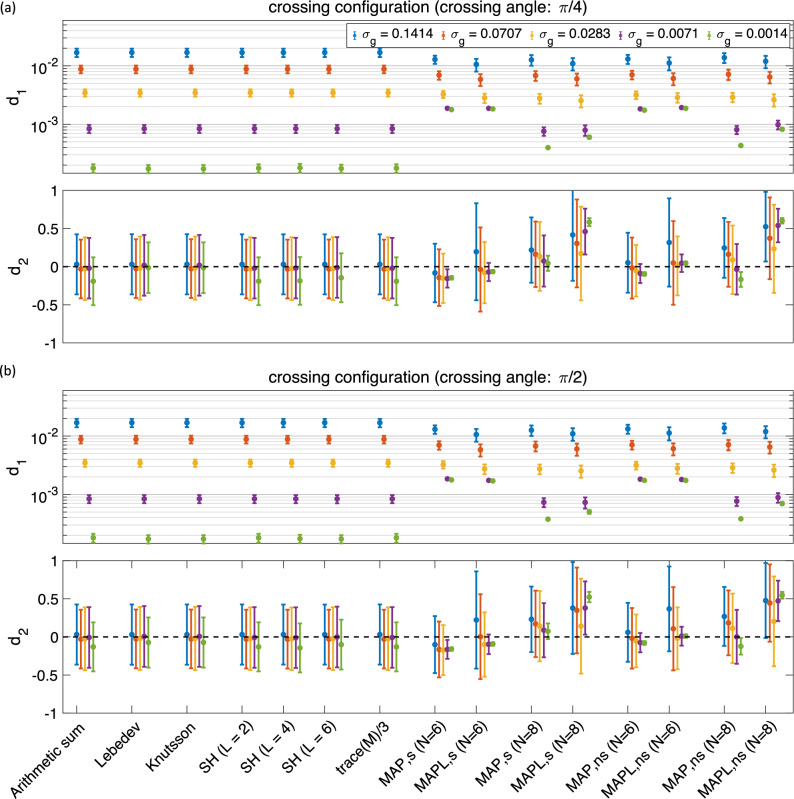
The mean and std of the $d_1$ and $d_2$ measures using different methods for 344 samples in the presence of crossing configuration with (**a**) $\pi /4$ and (**b**) $\pi /2$ radians crossing angle (the y-axis in $d_1$ is scaled logarithmically). The ‘s’ and ‘ns’ correspond to the shelled and non-shelled point sets, respectively. Different colors (blue, red, yellow, purple, green) show the results in different noise levels ($\sigma _g = 0.1414, \,0.0707, \,0.0283, \,0.0071,\, 0.0014$).

**Figure 5:**
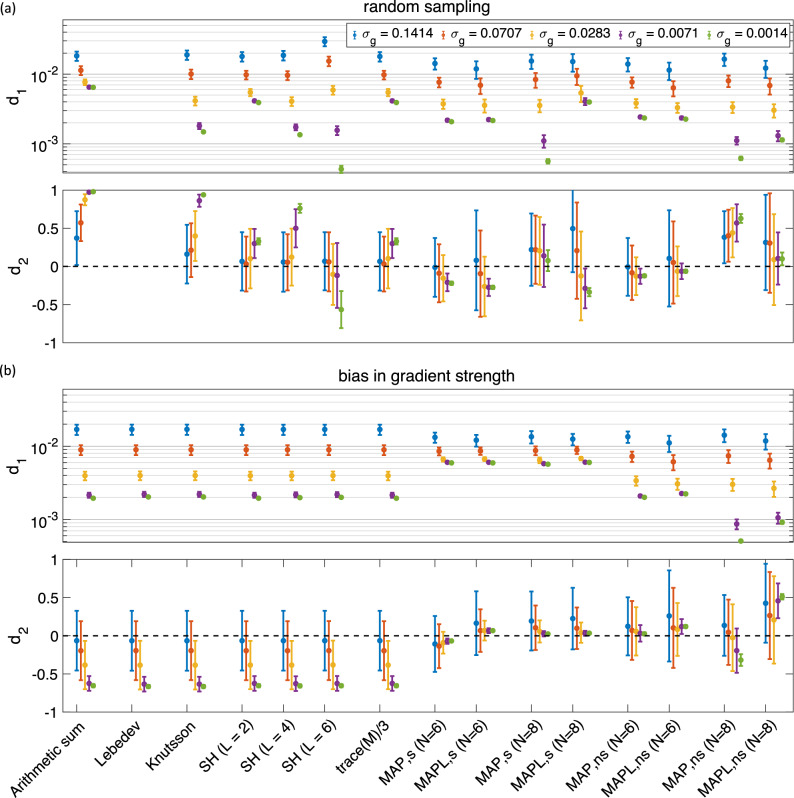
The mean and std of the $d_1$ and $d_2$ measures for 344 (43$\times$8) samples using different methods for (**a**) random sampling scheme, and (**b**) bias in gradient strength (the y-axis in $d_1$ is scaled logarithmically). The ‘s’ and ‘ns’ correspond to the shelled and non-shelled point sets, respectively. Different colors (blue, red, yellow, purple, green) show the results in different noise levels ($\sigma _g = 0.1414,\, 0.0707,\, 0.0283,\, 0.0071,\, 0.0014$).

**Figure 6:**
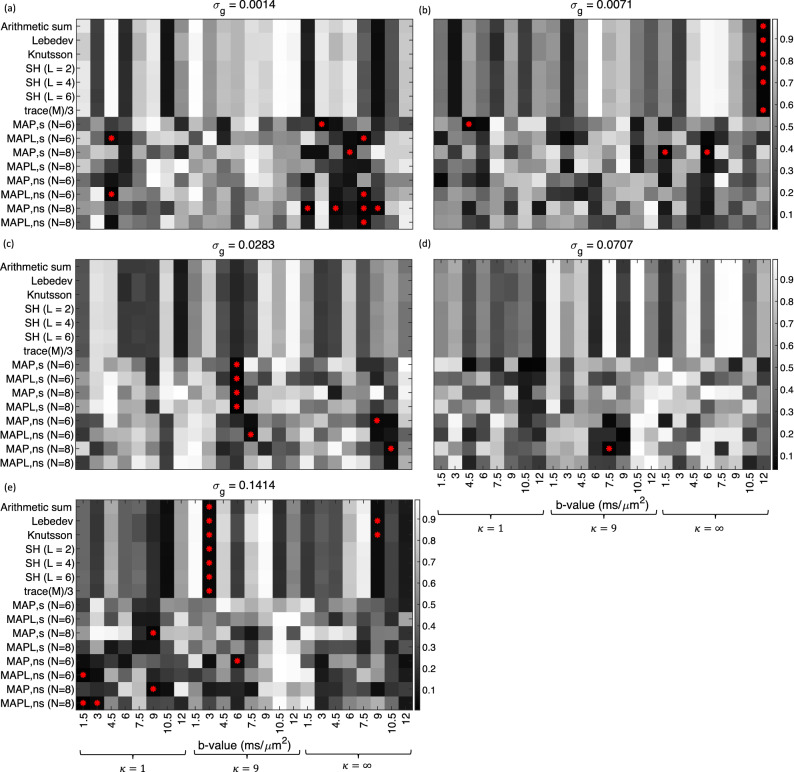
The *p*-values from the Anderson–Darling test^24^ on the orientationally averaged signal obtained from different approaches on 344 (43$\times$8) samples for each b-value and each dispersion value, $\kappa$. Panel (**a**) to (**e**) show the *p*-values for different noise levels from $\sigma _g = 0.0014$ in (**a**) to $\sigma _g = 0.1414$ in panel (**e**). The red asterisks illustrate the schemes that the orientationally averaged signal is not Gaussian.

**Figure 7:**
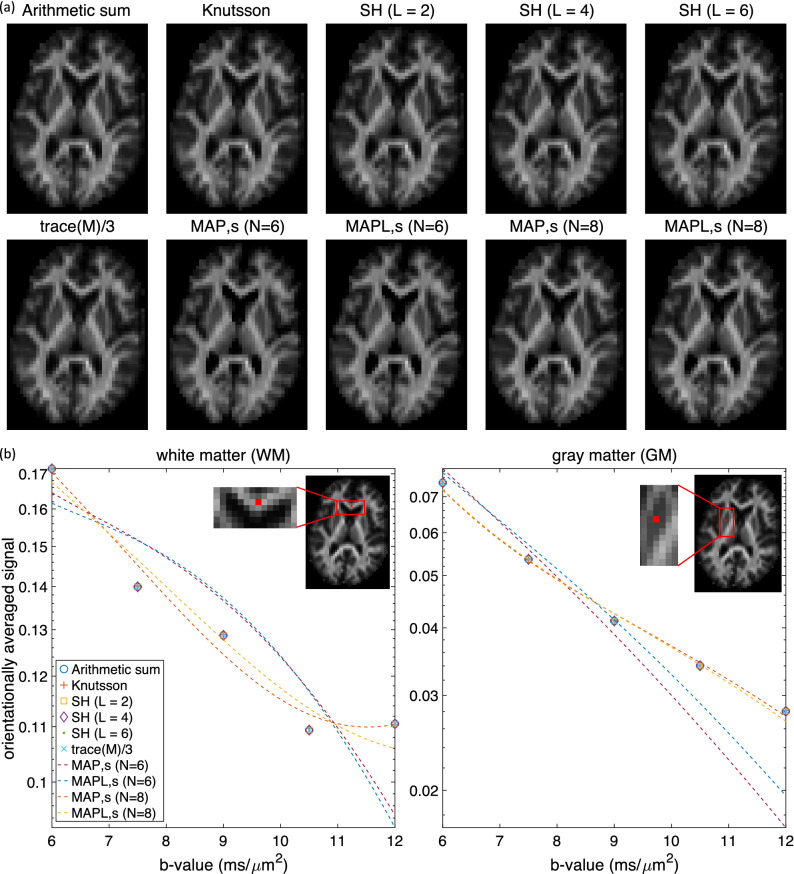
The estimated orientationally averaged signal using different methods for in vivo data on (**a**) an axial slice for $b = 6 \; {\rm ms}/\upmu {\rm m}^2$. (**b**) in a white matter (left plot) and a gray matter (right plot) voxel for $b = 6, \; 7.5, \; 9, \; 10.5$ and $12\,\,{\rm ms}/\upmu {\rm m}^2$ in shell-based methods and $b = 6, \; 6.1, \; 6.2, \; \ldots , \; 12\,\,{\rm ms}/\upmu {\rm m}^2$ in MAP-based approaches.

**Table 1:** The summary of powder-averaging techniques, different vector sets and different experiments conducted in this study.

	Estimation method	References
1- Shell-by-shell estimation	1- Arithmetic averaging	^6^
	2- Quadratures on the sphere (Lebedev)	^31^
	3- Representation of signal in spherical harmonic	^3^
	4- Representation of signal in Cartesian tensors	^41^
	5- Knutsson	^42^
2- Estimation from the entire 3D data	1- MAP-MRI	^44^
	2- MAP with Laplacian regularization	^51^

Point sets	Sampling scheme	References
Shelled	488 (61$\times$8) samples (Knutsson)	^21^
	344 (43$\times$8) samples (Lebedev)	^31^
	152 (19$\times$8) samples (Lebedev)	^31^
	344 (43$\times$8) random samples	
Non-shelled	488 samples (Knutsson)	^21^
	344 samples (Knutsson)	^21^
	152 samples (Knutsson)	^21^
	344 random samples	

Experiments	Sampling scheme	
Effect of noise and number of samples	488 (61$\times$8) samples^21^ with Gaussian noise	Figure 1a
	152 (19$\times$8)^21,31^ samples with Gaussian noise	Figure 1b
	344 (43$\times$8)^21,31^ samples with Gaussian noise	Figure 2a
	344 (43$\times$8)^21,31^ samples with Rician noise	Figure 2b
Effect of dispersion	344 (43$\times$8) samples^21,31^ with Gaussian noise	Figure 3
Crossing configuration	344 (43$\times$8) samples^21,31^ with Gaussian noise	Figure 4
Random sampling	344 (43$\times$8) samples with Gaussian noise	Figure 5a
Bias in gradient strength	344 (43$\times$8) samples^21,31^ with Gaussian noise	Figure 5b
Statistical analysis	344 (43$\times$8) samples^21,31^ with Gaussian noise	Figure 6
In vivo measurements	488 (61$\times$8) samples^21^	Figure 7

To factor out the effect of macroscopic anisotropy in diffusion MRI, i.e., to estimate the signal for the “powdered” structure, two approaches have been proposed: (i) taking the “isotropic component” of the signal^3,4^; this is typically achieved by representing the signal with a series of spherical harmonics and keeping the leading (zeroth order) term; and (ii) numerical computation of the orientational average of the diffusion-weighted signal profile^5,6^. Due to the emerging interest^7–16^ in employing the so-called “powder-averaged signal” for tissue characterization, here we consider the problem of estimating this quantity from data acquired using single diffusion encoding (SDE) protocols.

The accuracy of the powder-averaged signal depends on both the set of gradients employed in the data acquisition and the numerical method used to estimate the average. Regarding the former, different strategies have been proposed to optimize the sampling strategy, the most well-known and widely-used of which is the electrostatic repulsion algorithm^17^. Here, a uniform single-shell distribution of *q*-space sample points is found by minimizing the electrostatic energy of a system containing antipodal charge pairs on the surface of a sphere. Situations under which such single-shell diffusion sampling schemes are rotationally-invariant have been discussed previously^18,19^. We refer to the situation when point sets are distributed on the surface of a sphere as a “shelled” sampling scheme. In some applications, the sample points should be optimally distributed not just on a single sphere, but across three-dimensional *q*-space^20^. Knutsson et al.^21^ proposed a novel framework that extends the traditional electrostatic repulsion approach to generate optimized three-dimensional *q*-space sample distributions, by enabling a user-specific definition of the three-dimensional space to be sampled and associated distance metric. We refer to such sampling schemes as “non-shelled”.

The second important factor in orientational signal averaging is the method used to compute the powder-average, which is our focus in this study. Methods that perform brute-force estimation of the signal average and those that compute the “isotropic component” of the signal, (derived from signal representations), are compared. Our simulations feature varying levels of noise, to explore the performance of the different algorithms under different SNR levels.

## Results

### Effect of noise and number of samples

Figure 1a shows the results obtained from a sampling scheme with 488 (61$\times$8) samples for both shelled and non-shelled point sets in the presence of Gaussian noise. We note that in this sampling scheme, derived using Knutsson’s sampling algorithm^21^, we cannot compute the orientationally-averaged signal using Lebedev’s method because this weighted-averaging approach requires a particular set of sampling points and weights. The plots show the mean and standard deviation of two performance metrics (see Methods): $d_1$ (mean absolute difference between the computed and gold-standard orientational average, derived over all 3 orientational dispersions and b-value shells) and $d_2$ (the Pearson correlation between the (signed) difference in computed and gold-standard orientational-average, and the b-value).

Under noisy conditions, MAP-based methods outperform the shell-by-shell estimates, and the regularization employed in MAPL method evidently yields a further reduction in $d_1$. At low noise levels, for $\sigma _g \le 0.0071$, $d_1$ is very small for all methods, though slightly elevated for MAP and MAPL methods with $N_{\max} = 6$. The mean values of $d_2$ (which quantifies the *b*-dependent bias) are generally lower in shell-by-shell estimates. However, the standard deviation of $d_2$ is large in almost all the shell-based methods, even at the lowest noise level considered here ($\sigma _g = 0.0014$), which is likely a side-effect of treating shells independently in the averaging schemes. However, the standard deviation of $d_2$ is substantially reduced in MAP-based methods as the noise level decreases. As far as bias is concerned, MAP performs better than MAPL in most cases. MAP-based methods can also be used for interpolating the signal between the b-values (for more details see Supplementary Fig. S3 online).

Figure 1b illustrates the results for the sampling schemes with 152 ($19 \times 8$) samples in the presence of Gaussian noise. Here, Lebedev and Knutsson methods have the lowest error, $d_1$, when the noise level is very low ($\sigma _g$), for the other noise levels the performance of all the methods is similar. $d_2$ is close to zero for arithmetic sum in almost all noise levels while $d_2$ is mostly negative for the lowest noise level ($\sigma _g = 0.0014$) in most of the methods.

Figure 2a shows the results from the 344 ($43\times 8$) Lebedev sampling scheme in the presence of Gaussian noise (For a detailed representation of the orientationally-averaged signal over different b-values see Supplementary Fig. S2 online). Figure 2b shows the results obtained from the same 344 ($43 \times 8$) Lebedev sampling scheme in the presence of Rician noise. Clearly, when magnitude data are used and no bias correction scheme is employed, the Rician noise floor results in a significant bias ($d_2$) in the signal that needs to be corrected^22^. Doing so would also reduce the error, $d_1$, in the estimation of powder average signal. Given that methods exist to transform the distribution of the magnitude signal to Gausian^23^, we based our conclusions on the results obtained from simulated data assuming that a Gaussian signal distribution of the orientational averages.

Comparing the results from 488 ($61\times 8$) samples as shown in Fig. 1a, 344 ($43\times 8$) samples in Fig. 2a, and 152 ($19 \times 8$) samples in Fig. 1b, the errors and biases (and their spreads) increase as the number of samples is decreased as expected. In the case of 152 samples and at the lowest level of noise ($\sigma _g = 0.0014$), Lebedev, Knutsson, SH (L = 4 and 6), and MAPL for shelled and non-shelled samplings with $N_{\max} = 6$ lead to significant biases.

### Effect of dispersion

Figure 3 shows the impact of orientational dispersion on the orientational-averages. We used the (Lebedev-derived) set of 344 ($43 \times 8$) sample points and estimated $d_1$ and $d_2$ measures using the Knutsson, MAP and MAPL ($N = 6$ and 8) approaches. When $\kappa$ is large, there is little dispersion while reducing $\kappa$ increases the dispersion. The error ($d_1$) and bias ($d_2$) are about the same across $\kappa$ values, with slight improvement in $d_2$ as the orientational dispersion increases (i.e. $\kappa$ decreases).

### Crossing fibre configuration

Figure 4 shows the effect of crossing fibre configurations on the estimated orientationally-averaged signal for two different crossing angles $\pi /4$ and $\pi /2$ radians (Fig. 4a and b respectively). When the noise level is low, all methods have similar error $d_1$, when the noise level is increased, MAP-based methods work better than the shell-based methods.

### Non-uniform distribution of gradient directions

The effect of non-uniform distribution of gradient directions (derived in this case with a random sampling) is illustrated in Fig. 5a. When the noise level is high ($\sigma _g = 0.1414$ and $\sigma _g = 0.0707$), the error, $d_1$, is similar in all orientation averaging methods apart from the arithmetic averaging which has a slightly higher error. When the noise level is high, SH(L=6) gives the largest value of $d_1$, while the bias, $d_2$, varies across noise levels for all techniques. This result underlines that careful choice of sampling points, is just as important as the orientation averaging technique in obtaining an accurate orientationally averaged signal with a low error ($d_1$) and bias ($d_2$).

### Effect of bias in gradient strength

Figure 5b shows the effect of bias in gradient strength on the orientationally-averaged signal for different methods. The amount of error, $d_1$, is similar in all methods while the bias, $d_2$, is more pronounced (more negative in this case) in shell-by-shell approaches compared to MAP-based techniques.

### Statistical analysis

We investigated the normality of the orientational-averages via the Anderson–Darling test^24^ and the results are provided in Fig. 6. Different techniques give mostly Gaussian results (*p*-values are greater than 0.05). Occasional non-normality is detected (shown by the red asterisks) which may be due to noise, but almost twice as many occur with MAP-based methods compared with non-MAP-based methods. The *p*-values suggest that shell-by-shell techniques are consistent for a given set of signal values, but different flavors or orders of MAP are not consistent. The *p*-value obtained for a given technique (shell-by-shell or MAP) exhibits dependence on experimental parameters.

### In vivo data

Figure 7a shows the estimated orientationally-averaged signal at $b = 6 \; ms/ \mu m^2$ for one axial brain slice using different methods. The maps look visually similar. In Fig. 7b the orientationally-averaged signals for different b-values ($b = 6, \; 7.5, \; 9, \; 10.5, \; 12 ms/ \mu m^2$) for a white matter and a gray matter voxel are illustrated. The non-MAP-based averages all super-impose pretty perfectly while the MAP-based results are different. A likely reason for this differences is that in the MAP estimates, we enforce constraints that make the average propagator nonnegative. It is also to be noted that when $N_{max}=6$, the regularization associated with MAP-MRI is quite strong. This may prevent overfitting in very noisy situations while suppressing some relevant features in others. These findings are consistent with those on simulations.

## Discussion

Our analyses demonstrate that the choice of both the q-space sampling scheme and the numerical method for powder-averaging affect the estimated orientationally-averaged signal.

Our comparisons illustrated the relative performance of various methods subject to different noise levels and sampling schemes. Although our simulations are limited in terms of the complexity of the signal, we believe the simulated scenario is representative of commonly encountered signal profiles. The analyses can be repeated in a similar manner for investigations targeting specific features (e.g. power laws^25^) in the signal.

Methods for averaging the signal on a single shell that are based on taking the ‘isotropic component’ of the signal yield weighted-averages of the original signal samples. The corresponding weights are given through Eqs. (8)–(9) and (13). The magnitude-valued MRI data are Rician distributed and our simulations show that without correcting for Rician noise, the powder-averaged signal is far from the ground truth especially when the signal-to-noise ratio (SNR) is low. The SH and trace(M)/3 approaches lead to similar results as arithmetic averaging, with the difference that the SH approach with high order (see results for $L = 4$ or $L = 6$) is preferred over arithmetic averaging when the number of directions is low (see result for 19 directions).

Non-optimal acquisition schemes such as non-uniform distributions of gradient directions or (unccorrected) biases in gradient strength cause error in the orientationally-averaged signal. As gradient strengths are pushed higher and higher^26,27^ maintaining gradient linearity becomes more and more challenging^28^, and having truly shelled acquisitions becomes infeasible. MAP-MRI is particularly well suited to these situations as it provides a representation of the diffusion MRI signal over the three-dimensional gradient sampling space.

Various works in the literature, fitting multi-compartment models to derive microstructural parameters, have relied on information captured in the powder-averaged signal (e.g.^16,29^). Employing an optimal q-space sampling scheme and robust direction-averaging method could significantly reduce the bias in parameter estimates from these models (see Supplementary Fig. S4 online). The MAPL technique, which introduces regularization in lieu of constrained estimation, has lower error ($d_1$) compared to MAP when there is a high level of noise but it leads to higher bias ($d_2$) compared to original MAP. In the in vivo data, there is some difference between the estimates from MAP and others. A likely reason is that in the MAP estimates, we enforce constraints that make the average propagator nonnegative. It is also to be noted that when $N_{max}=6$, the regularization associated with MAP-MRI is quite strong. This may prevent overfitting in very noisy situations while suppressing some relevant features in others. These findings are consistent with those on simulations. We note that the very recent formulation^30^ of the MAP method with hard constraints on the positivity of the estimated propagator (MAP+) could further improve the accuracy of the estimates.

## Methods

In this Section, we review the details of six different approaches (four of which are sub-types of “weighted averaging”) to estimating the powder-averaged diffusion-weighted MRI signal (Table 1). We also detail the Monte Carlo simulations, evaluation criteria for the comparisons of schemes, point sets used and the experiments conducted.

### Approaches for powder averaging

#### Arithmetic averaging

The simplest technique for powder-averaging is to distribute the samples as uniformly as possible over a sphere, and then compute the arithmetic mean of those measurements on that particular sphere^6^. In this scheme, the powder averaged signal for a given b-value (${\bar{S}}$) is estimated through

1 $$\begin{aligned} {\bar{S}} = \frac{1}{n_{\rm dir}}\sum _{i = 1}^{n_{\rm dir}}S_i \ , \end{aligned}$$

where $n_{\rm dir}$ is the number of gradient directions and $S_i$ is the signal along the *i*th gradient direction. However, perfectly uniformly-distributed sets of directions are not readily attainable and therefore other methods have been proposed to overcome this problem.

#### Weighted averaging

In most diffusion-weighted sampling schemes, weighted averaging can be used to account for the non-uniformity of the gradient directions. For each b-value, the powder averaged signal (${\bar{S}}$) is

2 $$\begin{aligned} {\bar{S}} = \frac{\sum _{i = 1}^{n_{\rm dir}}w_i\, S_i}{\sum _{i = 1}^{n_{\rm dir}}\, w_i} \end{aligned}$$

where $w_i$ are the weights corresponding to the signal along the *i*th gradient direction. We considered four techniques that enable the determination of an optimal set of weights, $w_i$, for the estimation of the orientationally-averaged signal. (Lebedev, Knutsson, SH, and trace(M)/3 are all sub-types of weighted averaging).

**Quadratures on the sphere (Lebedev):** Lebedev quadrature evaluates integrals over the unit sphere, i.e.,

3 $$\begin{aligned} I = \int _0^{2\pi } \int _0^\pi f(\theta ,\phi ) \, \sin \theta \, {\rm d}\theta \, {\rm d}\phi \end{aligned}$$

as an approximation

4 $$\begin{aligned} I \approx \sum _{i=1}^N f(\theta _i, \phi _i) \, w_i \equiv Q[f] \end{aligned}$$

where the points ($\theta _i$, $\phi _i$) and weights $w_i$ are estimated for different *N* using the algorithm in^31^. Q[f] is the quadrature of the exact integral. Similar to the one-dimensional Gauss quadratures, the nodes, ($\theta _i$, $\phi _i$), and the weights can be determined simultaneously. A constraint is imposed to integrate all spherical harmonics up to degree *p*. This leads to a system of nonlinear equations, which can be solved to provide the optimal nodes and weights. This idea stems from the work by Sobolev^32^.

Lebedev built a set of quadratures and the set of non-linear equations are solved for degrees up to $p = 131$^33–37^, yielding one of the most commonly-used quadratures for integration over the sphere.

In this study, we employ this technique by setting $f(\theta ,\phi )$ to be the signal profile at a particular *b*-value. As this technique requires the value of the function to be evaluated along specific directions, the set of gradient directions has to be chosen accordingly, i.e., the technique cannot be readily employed with commonly-available diffusion MRI sampling protocols.

**Representation of the single-shell signal in a series of spherical harmonics:** Any square-integrable function, $F(\hat{\mathbf {x}})$, defined over the unit sphere, can be represented in a spherical harmonic basis through the expansion

5 $$\begin{aligned} F(\hat{\mathbf {x}}) = \sum _{k=0}^\infty \sum _{m=-k}^k f_{km} \, Y_k^m (\hat{\mathbf {x}}) \ , \end{aligned}$$

where *k* and *m* indicate the order of the spherical harmonics function denoted by $Y_k^m (\hat{\mathbf {x}})$. The coefficients are given by

6 $$\begin{aligned} f_{km} = \int F(\hat{\mathbf {x}}) \, Y_k^m (\hat{\mathbf {x}})^* \,{\mathrm{d}}\hat{\mathbf {x}} \ . \end{aligned}$$

where ’$^*$’ denotes the complex conjugate. If $F(\hat{\mathbf {x}})$ is rotationally-invariant, i.e., independent of $\hat{\mathbf {x}}$, all coefficients except $f_{00}$ vanish.

Spherical harmonics can be used to represent the diffusion signal profile at a fixed *b*-value^38^ by terminating the series at a finite value $k_{\max}=L$. In matrix form, such a representation can be written as

7 $$\begin{aligned} {\mathbf {s}} = \mathbf {Ya} \ , \end{aligned}$$

where $\mathbf {s}_{n_{\rm dir}\times 1}$ is the vector of diffusion-weighted signals, $\mathbf {Y}_{n_{\rm dir}\times n_{\rm coeff}}$ is the matrix of spherical harmonics for $n_{\rm coeff}=(L+1)(L+2)/2$ number of coefficients, and $\mathbf {a}_{n_{\rm coeff} \times 1}$ is the vector of coefficients, which can be estimated using

8 $$\begin{aligned} {\mathbf {a}} = ({\mathbf {Y}}^\intercal {\mathbf {Y}})^{-1}{\mathbf {Y}}^\intercal {\mathbf {s}} \ . \end{aligned}$$

As mentioned earlier, an estimate of the powder-averaged signal is obtained by taking the signal’s “isotropic component” in its irreducible representation^3^. When adopted to SDE acquisitions on a single shell, the orientationally-averaged signal is given simply by the coefficient corresponding to the $k=m=0$ term of the series, or more explicitly,

9 $$\begin{aligned} {\bar{S}} = a_0 Y_0^0 = a_0/\sqrt{4\pi } \ , \end{aligned}$$

where we employ the convention that $Y_0^0 = 1/\sqrt{4\pi }$^39^.

Note that Eqs. (8) and (9) suggest that the powder-average estimate using this scheme is also a weighted average of the signal values, albeit without any constraint on the selection of gradient directions.

**Representation of the single-shell signal using Cartesian tensors:** The diffusion-weighted signal profile at a fixed b-value can also be represented by the following equation:

10 $$\begin{aligned} S({\hat{\mathbf {u}}})={\hat{\mathbf {u}}}^\intercal {\mathbf {M}} {\hat{\mathbf {u}}}\ , \end{aligned}$$

where ${\hat{\mathbf {u}}}$ is the unit vector denoting the gradient direction and ${\mathbf {M}}$ is a $3\times 3$ symmetric positive definite matrix. In this representation, the orientationally-averaged signal is given by

11 $$\begin{aligned} {\bar{S}} = \mathrm {Tr}({\mathbf {M}})/3 \ . \end{aligned}$$

Considering that ${\mathbf {M}}$ is symmetric, we can cast the problem in the matrix form

12 $$\begin{aligned} {\mathbf {s}} = \mathbf {U} {\mathbf {m}} \ , \end{aligned}$$

where ${\mathbf {U}}_{n_{\rm dir} \times 6}$ is the matrix whose each row is given in terms of the corresponding gradient direction $[u_{x}^2, u_{y}^2, u_{z}^2, u_x u_y, u_x u_z, u_y u_z]$, and ${\mathbf {m}} = [M_{xx}, M_{yy}, M_{zz}, M_{xy}, M_{xz}, M_{yz}]^\intercal$.

The powder-averaged signal (11) can thus be estimated via

13 $$\begin{aligned} {\bar{S}} = \mathbf {h}^\intercal {\mathbf {m}} = \mathbf {h}^\intercal ({\mathbf {U}}^ \intercal {\mathbf {U}})^{-1} {\mathbf {U}}^\intercal {\mathbf {s}} \ , \end{aligned}$$

where $${\mathbf {h}} = [1/3, 1/3, 1/3, 0,0,0]^\intercal$$.

As discussed in the context of representing the apparent diffusivity profiles^40,41^, from a conceptual point-of-view, the representation (10) is equivalent to the representation of the signal in terms of a series of spherical harmonics terminated at $k=2$. However, we include it here as a separate scheme to assess potential numerical differences between the methods. We also note that representations in terms of higher order Cartesian tensors equivalent to the series of spherical harmonics terminated at higher orders can be formulated^40^, but excluded here for brevity.

**Knutsson:** Following from the spherical harmonic representation of the signal, the rotational variance of a set of unit vectors $\hat{\mathbf {u}}_i$, with $i=1\ldots {n_{\rm dir}}$, can be analyzed using spherical harmonics in the following way^42,43^. Consider a weighted sampling function of the form

14 $$\begin{aligned} G(\hat{\mathbf {x}}) = \sum _{i=1}^{n_{\rm dir}} w_i \, \delta (\hat{\mathbf {x}}-\hat{\mathbf {u}}_i) \ . \end{aligned}$$

The coefficients of this function in the spherical harmonic basis is given by

15 $$\begin{aligned} g_{km} = \sum _{i=1}^{n_{\rm dir}} w_i \, Y_k^m(\hat{\mathbf {u}}_i)^* \ . \end{aligned}$$

For rotationally-invariant sampling, these coefficients would obey $g_{km}\propto \delta _{k0}\delta _{m0}$. Let us denote by $\mathbf {g}_0$ the vector of coefficients having non-zero value only when $k=m=0$. An optimal vector of weights, $\mathbf {w}_0$, can be obtained using the expression^42^

16 $$\begin{aligned} \mathbf {w}_0 = \mathrm {argmin}[(\mathbf {Bw}-\mathbf {g}_0)^\intercal \mathbf {V}(\mathbf {Bw}-\mathbf {g}_0)] = (\mathbf {B}^\intercal \mathbf {VB})^{-1}(\mathbf {VB})^\intercal \mathbf {g}_0 \ , \end{aligned}$$

where $\mathbf {B}$ is the matrix of the spherical harmonics basis sampled at the orientation $\hat{\mathbf {u}}_i$, and $\mathbf {V}$ is a diagonal matrix containing the weights for each spherical harmonic^42^. $\mathbf {V}$, which corresponds to $\mathbf {W}_k^2$ in equation (4) in^42^, is used to set the importance of obtaining the specified response for different spherical harmonic basis functions which relate to the expected signal content of different spherical harmonics in the measurement. This depends on the tissue as well as on the b-value. We found that taking $\mathbf {V}$ to be a diagonal matrix with diagonal elements given by $(1+k^2/36)^{-1}$ provides adequate distribution of weights to respective terms of the series for typical signal decay profiles within the brain parenchyma. The size of the matrices depends on the number of directions. In the implementation, the maximum order *k* was taken to be 18, 14, and 10 for 61, 43, and 19 directions, respectively. We note that using this procedure, arithmetic averaging could be justified only for those sets of unit vectors that lead to equal weights, $w_i$. Such sets of unit vectors are not attainable for all but a few very special cases of ${n_{\rm dir}}$. Equation (16) and the result of spherical harmonic representation are the same (with ${\mathbf {B}} = {\mathbf {Y}}^\intercal$) if the weighting function ${\mathbf {V}}$ in Eq. (16) is unity for degree 1 to *L* and zero for higher *L*.

#### MAP-MRI

Above, we described several techniques with which the orientationally-averaged signal can be estimated from single-shell data wherein the data points are collected by repeatedly applying gradients along different directions while keeping the b-value fixed. In most applications involving the powder-averaged signal (${{\bar{S}}}$), one is interested in characterizing the dependence of the signal on the b-value. This can be accomplished by acquiring data on multiple shells and repeatedly applying one of the above methods on each shell. Alternatively, one can employ a representation of the signal on the entire three-dimensional space sampled by the gradient vector and compute its “isotropic component” similar to what was done above for the spherical harmonics representation. MAP-MRI^44^ is a powerful representation, which was shown to not only reproduce the diffusion-weighted signal over the three-dimensional space (see the result of ISBI challenge 2020 as an example^45–47)^, but also provide accurate estimates of scalar measures from datasets including heavily diffusion-weighted acquisitions^48^. One scalar index derived from such sampling is the propagator anisotropy (PA) whose formulation involves the estimation of the isotropic component of the propagator^44^, which can be adopted for estimating the orientationally-averaged signal as indicated before. This is accomplished by using the formulation of MAP-MRI in spherical coordinates^44,49^. In Cartesian coordinates, the formulation of MAP-MRI follows, in a straightforward manner, from its one-dimensional version^50^ that features Hermite polynomials and allows for anisotropic scaling of the basis functions. When expressed in spherical coordinates, the following equation is used for this purpose (equation (58) in^44^):

17 $$\begin{aligned} S(q, {\hat{\mathbf {q}}}) = \sum _{N=0}^{N_{\max}} \sum _{j,l}\sum _{m=-l}^{l}\kappa _{jlm}\Xi _{jlm}(u_0, q, {\hat{\mathbf {q}}}) \ , \end{aligned}$$

where $j\ge 1$, $l\ge 0$ and $2j + l = N + 2$, $u_0$ is a scalar related to the width of the basis functions, and *q* and ${\hat{\mathbf {q}}}$ are the magnitude and direction of the wavevector, which is proportional to the gradient vector. The basis function $\Xi _{jlm}(u_0,q,{\hat{\mathbf {q}}})$ is given by

18 $$\begin{aligned} \Xi _{jlm}(u_0,q, {\hat{\mathbf {q}}})= \sqrt{4\pi }i^{-1}(2\pi ^2u_0^2q^2)^{l/2}e^{-2\pi ^2u_0^2q^2} L_{j-1}^{l+1/2}(4\pi ^2u_0^2q^2)Y_{l}^m({\hat{\mathbf {q}}}) \ , \end{aligned}$$

where $L_{k}^{\alpha }(.)$ is the associated Laguerre polynomial and $Y_{l}^m({\hat{\mathbf {q}}})$ is the spherical harmonic.

The isotropic part of the diffusion-weighted signal is the powder-averaged signal, which is obtained by setting $l = m = 0$ and $j = 1+N/2$:

19 $$\begin{aligned} {\bar{S}} = \sum _{N=0}^{N_{\max}}\kappa _{(1+N/2)00}\,\Xi _{(1+N/2)00}(u_0,\mathbf {q}) . \end{aligned}$$

In this study, we employed two versions of the MAP-MRI technique: (i) its original formulation by Özarslan et al., in which the positivity of the propagator is enforced over a large domain in displacement space^44^; and (ii) a later formulation called MAPL introduced by Fick et al.^51^ in which a Laplacian regularization is employed instead of the constraints, similar to what was done in the corresponding one-dimensional problem^52^.

### Simulations

The noise-free diffusion-weighted signal at a b-value *b* when the gradients are applied along the unit vector ${\hat{\mathbf {u}}}$ was generated using the following equation:

20 $$\begin{aligned} S(b, {\hat{\mathbf {u}}}) = \int W({\hat{\mathbf {n}}},\hat{\varvec{\mu }})\, e^{-b {\hat{\mathbf {u}}}^\intercal \, {\mathbf {D}}( {\hat{\mathbf {n}}})\, {\hat{\mathbf {u}}}} \, {\text{d}}{\hat{\mathbf{n}}} \ , \end{aligned}$$

where ${\mathbf {D}}( {\hat{\mathbf {n}}})$ is an axisymmetric, prolate tensor oriented along ${\hat{\mathbf {n}}}$ with eigenvalues $D^{\mid \mid } = 1 \,\mu m^2/ms$, and $D^{\perp } = 0.14 \, \mu m^2/ms$. The orientation distribution function, $W({\hat{\mathbf {n}}},{\hat{\varvec{\mu }}})$ is taken to be a Watson distribution function given by

21 $$\begin{aligned} W({\hat{\mathbf {n}}},{\hat{\varvec{\mu }}}) = M(1/2, 3/2/ \kappa )^{-1} e^{\kappa ({\hat{\varvec{\mu }}} \cdot {\hat{\mathbf {n}}})^2} \ , \end{aligned}$$

where *M* is the confluent hypergeometric function, ${\hat{\varvec{\mu }}}$ is the mean direction, taken to be $(0.4,\,0.6,\,-0.693)^\intercal$ and $\kappa$ is the concentration parameter. When $\kappa$ is small, for example, $\kappa = 1$ we have high orientation dispersion (i.e, we have a fat orientation distribution function (ODF)) and when $\kappa$ is large, for example, $\kappa = 64$, the orientation dispersion is small and the ODF is sharp. In our simulation, we used $\kappa =$ 1, 9, and $\infty$ (a signal without dispersion or Dirac delta ODF). Irrespective of the orientation distribution function, the ground truth orientationally-averaged signal is given by^12,53^

22 $$\begin{aligned} {\bar{S}}_{\rm gt}(b)=\frac{\sqrt{\pi }{e}^{-bD^{\perp }}}{2}\frac{{\rm erf}(\sqrt{b(D^{\mid \mid }-D^{\perp })})}{\sqrt{b(D^{\mid \mid }-D^{\perp })}} \ . \end{aligned}$$

The multi-shell data were computed at the b-values $b = 0,\, 1.5,\, 3,\, 4.5,\, 6,\, 7.5,\, 9,\, 10.5$, and $12 \, {\rm ms}/\upmu {\rm m}^2$ and with $\Delta = 43.1 \, ms$ and $\delta = 10.6 \, ms$. For the above parameters and with $b = q^2(\Delta - \delta /3)$, we expect the following signal values: ${{\bar{E}}}(b) =$ 1, 0.5640, 0.3541, 0.2386, 0.1682, 0.1221, 0.0903, 0.0678, 0.0514. The noisy diffusion-weighted signal values are synthesised according to the following for Gaussian and Rician distributed signals, respectively:

23 $$\begin{aligned} {S_{\rm ng}}(b, {\hat{\mathbf {u}}})= & {} {S_{\rm gt}}(b, {\hat{\mathbf {u}}}) + {N_{\rm r}}(0, {\sigma_{\rm g}}) \end{aligned}$$

24 $$\begin{aligned} {S_{\rm nr}}(b, {\hat{\mathbf {u}}})= & {} \sqrt{({S_{\rm gt}}(b, {\hat{\mathbf {u}}})+ {N_{\rm r}}(0, {\sigma_{\rm g}}))^2 + N_i(0, {\sigma_{\rm g}})^2} \ , \end{aligned}$$

where $N_r(0,{\sigma_{\rm g}})$ and $N_i(0,{\sigma_{\rm g}})$ are the normal distributed noise in, respectively, the real and imaginary images with a standard deviation of ${\sigma_{\rm g}}$. Here, we simulate the noisy signal with ${\sigma_{\rm g}} =$ 0.1414, 0.0707, 0.0283, 0.0071, and 0.0014.

### In vivo data

One healthy participant who showed no evidence of a clinical neurologic condition was scanned with the approval of the Cardiff University School of Psychology Ethics Committee. The acquisition was carried out in accordance with relevant guidelines and regulations and informed consent was obtained from the participant. Diffusion-weighted images were acquired on a 3T Connectom MR imaging system with 300 mT/m gradients (Siemens Healthineers, Erlangen, Germany). The protocol comprised 10 $b=0$ and 8 non-zero shells ($b=1, \, 2, \, 3, \, 4.5, \, 6, \, 7.5, \, 9, \, 10.5, \, 12 \; {\rm ms}/\upmu {\rm m}^2$) along ($31, \, 31, \, 31, \, 31, \, 61, \, 61, \, 61, \, 61, \, 61$) directions. The 61 and 31 orientations were optimized based on^21^. Forty-two axial slices with 3*mm* isotropic voxel size and a 70$\times$70 matrix size, TE = 88 ms, TR = 5200 ms, partial Fourier factor = 6/8, were obtained. The total acquisition time was around 40 minutes. The data of this study can be found at 10.17035/d.2021.0136784104.

The diffusion weighted images were corrected for Rician bias^20^, and Gibbs ringing^54^. Eddy currents and subject motion were corrected using FSL EDDY^55^. We normalized the orientationally-averaged signal based on the b = 0 $s/mm^2$ signal in each voxel.

### Evaluation criteria

We utilized two measures $d_1$ and $d_2$ to quantify the fidelity of the orientationally-averaged signal. $d_1$ shows the absolute error between the estimated signal and the ground truth. For the correlation between the bias, $\epsilon _j$, and the b-values, the Pearson’s correlation coefficient ($d_2$) was used.

25 $$\begin{aligned} d_1= & {} \frac{1}{24} \sum _{j = 1}^8 \sum _{k=1}^3|\bar{S}_{\rm est}(b_j,\kappa _k) - {{\bar{S}}}_{\rm gt}(b_j)| \end{aligned}$$

26 $$\begin{aligned} \epsilon _j= & {} \frac{1}{3} \sum _{k=1}^3 \bar{S}_{\rm est}(b_j,\kappa _k) - {{\bar{S}}}_{\rm gt}(b_j) \end{aligned}$$

27 $$\begin{aligned} d_2= & {} \text {Pearson's correlation coefficient}(b_j, \epsilon _j) \ , \end{aligned}$$

where ${{\bar{S}}}_{\rm est}$ and ${{\bar{S}}}_{\rm gt}$ are, respectively, the estimated powder average signal and the ground truth values.

We are interested to know if there is correlation between the error and b-value and $d_2$ essentially evaluates this. The value of $d_2$ can only be interpreted by simultaneously considering $d_1$ because there are some cases that the estimates have a mean value close to the ground truth with a large variance, in this situation $d_2$ may be very small and it cannot represent the amount of error if it is considered alone.

### Point sets

#### Shelled point sets

Knutsson’s sampling scheme was used to generate point sets with 61 distinct gradient orientations^21^ and Lebedev’s method^31^ to produce sets with 43 and 19 orientation. The signal values were estimated at 8 b-values along each orientation. Therefore we have (Ydir $\times$ Zb) samples for each scheme where Ydir is the number of orientations per shell and Zb is the number of b-shells. For each sampling scheme, the same number of *S*(0) signal values was considered (i.e. 61 *S*(0) signal values for Knutsson’s method^21^ and 43 and 19 *S*(0) signal values for Lebedev’s method^31^).

#### Non-shelled point sets

The three-dimensional (non-shelled) gradient orientation sets were generated using Knutsson’s method for 488, 344, and 152 gradient directions^21^ matching the total number of samples ($61\times 8$, $43\times 8$, and $19\times 8$) in the shelled point sets. For 488, 344, and 152 sampling schemes 61, 43, and 19 *S*(0) signal values were considered. Table 1 and Fig. S1 summarise these point sets.

### Experiments

We conducted three different experiments to investigate the effect of encoding scheme and powder-averaging technique on the estimated orientationally-averaged signal.

#### Effect of noise and number of directions

For designing an experiment, one of the important factors is minimizing the total acquisition time especially for in vivo applications. However, at the same time, the set of measurements should provide sufficient information for robust model fitting. The acquisition should therefore be optimized to provide maximum information per unit time. In this work, we compared four different sets of sampling vectors, 61$\times$8^21^, 43$\times$8^31^, 19$\times$8^31^, and random 43$\times$8 point sets (shelled and non-shelled^21^) with different noise levels to investigate the effect of encoding scheme and noise on the estimated powder-averaged signal.

#### Effect of dispersion

The powder-averaged signal should provide rotationally-invariant tissue measures. In addition to the sampling scheme mentioned in the previous section the amount of actual orientational dispersion in the underlying structure will also affect the accuracy of the orientationally-averaged signal estimates. In order to investigate the effect of dispersion, we tested the orientationally averaged signal for three different dispersion parameters, $\kappa = 1, \; 9$ and $\infty$.

#### Crossing configuration

We performed simulations to examine the effect of fiber crossings on the orientationally averaged signal obtained via different methods. The crossing is modeled as multi-Watson with the same tensor shape and crossing angle of $\pi /4$ and $\pi /2$ radians. The signal for the crossing configuration is given by:

28 $$\begin{aligned} S(b, {\hat{\mathbf {u}}}) = 0.5 \int W({\hat{\mathbf {n}}},\hat{\varvec{\mu }}_1)\, e^{-b {\hat{\mathbf {u}}}^\intercal \, {\mathbf {D}}( {\hat{\mathbf {n}}})\, {\hat{\mathbf {u}}}} \, {\mathrm{d}}{\hat{\mathbf {n}}} \ + 0.5 \int W({\hat{\mathbf {n}}},{\hat{\varvec{\mu }}}_2)\, e^{-b {\hat{\mathbf {u}}}^\intercal \, {\mathbf {D}}( {\hat{\mathbf {n}}})\, {\hat{\mathbf {u}}}} \, {\mathrm{d}}{\hat{\mathbf {n}}} \ , \end{aligned}$$

where ${\hat{\varvec{\mu }}}_1 = {\hat{\varvec{\mu }}}$ is the first direction and ${\hat{\varvec{\mu }}}_2$ is the second direction defined as $(-0.384,\,-0.576,\,-0.721)^\intercal$ and $(-0.011,\,-0.017,\,-0.999)^\intercal$ for $\pi /2$ and $\pi /4$ crossing angles, respectively. As the same parameters (tensor shape and Watson distribution function) are used for both directions, the orientationally averaged signal is equal to the one obtained from a no-crossing configuration.

#### Non-uniform distribution of gradient directions

Two sets of non-uniformly distributed points were generated to investigate the effect of non-uniform distribution of gradient directions on the orientationally-averaged signal. The first set (shelled point set) contains 43 samples that are randomly selected from a 102 uniformly distributed points^21^. The second set of directions (non-shelled) includes 344 randomly generated point sets that are randomly distributed over q-space (random set of b-values with $b_{max} = 12 \; {\rm ms}/\upmu {\rm m}^2$) (see Supplementary Fig. S1 online).

#### Effect of bias in gradient strength

If there is bias in gradient strength, the acquired data might not be completely shelled even if it is collected using a shelled set of points (i.e. some data points are slightly inside or outside the shell instead of being perfectly on the surface of the shell). To simulate this situation, we added some random positive and negative values on the order of $10\%$ of the b-value to each shell on the shelled data and for the non-shelled data, this $10\%$ bias is considered at each b-value.

#### Statistical analysis

We performed the statistical analysis using the Anderson–Darling test on the orientationally averaged signal using different techniques. The test shows whether the orientationally averaged signal of 100 noise realization follows a Gaussian distribution for each b-value and each dispersion parameter, $\kappa$. Therefore the input to the test is a vector containing the 100 samples after orientation averaging using each method. Command ‘adtest’ in MATLAB was used for the statistical test which returns a test decision for the null hypothesis that the data come from a Gaussian distribution. The result of the test is 1 if the test rejects the null hypothesis at the $5\%$ significance level, and 0 otherwise. It also returns the corresponding *p*-value. The reason for testing the Gaussianity of the orientationally averaged signal is that normal distributed estimates offer advantages if there will be a subsequent fitting.

Table 1 summarizes different experiments conducted in this study. The code is available on github (https://github.com/maryamafzali/orientation_averaging).

## Supplementary Information

Supplementary Information 1.

## References

[1] Edén M (2003). Computer simulations in solid-state NMR. III. Powder averaging. Concepts Magn. Reson. Part A: Educ. J..

[2] Mitra PP, Sen PN (1992). Effects of microgeometry and surface relaxation on NMR pulsed-field-gradient experiments: Simple pore geometries. Phys. Rev. B.

[3] Özarslan E, Basser PJ (2008). Microscopic anisotropy revealed by NMR double pulsed field gradient experiments with arbitrary timing parameters. J. Chem. Phys..

[4] Özarslan E (2009). Compartment shape anisotropy (CSA) revealed by double pulsed field gradient MR. J. Magn. Reson..

[5] Jespersen SN, Lundell H, Sønderby CK, Dyrby TB (2013). Orientationally invariant metrics of apparent compartment eccentricity from double pulsed field gradient diffusion experiments. NMR Biomed..

[6] Kaden E, Kruggel F, Alexander DC (2016). Quantitative mapping of the per-axon diffusion coefficients in brain white matter. Magn. Reson. Med..

[7] Lasič S, Szczepankiewicz F, Eriksson S, Nilsson M, Topgaard D (2014). Microanisotropy imaging: Quantification of microscopic diffusion anisotropy and orientational order parameter by diffusion MRI with magic-angle spinning of the q-vector. Front. Phys..

[8] Szczepankiewicz F (2015). Quantification of microscopic diffusion anisotropy disentangles effects of orientation dispersion from microstructure: Applications in healthy volunteers and in brain tumors. NeuroImage.

[9] Lawrenz M, Finsterbusch J (2015). Mapping measures of microscopic diffusion anisotropy in human brain white matter in vivo with double-wave-vector diffusion-weighted imaging. Magn. Reson. Med..

[10] McKinnon ET, Jensen JH, Glenn GR, Helpern JA (2017). Dependence on b-value of the direction-averaged diffusion-weighted imaging signal in brain. Magn. Reson. Imaging.

[11] Özarslan E, Yolcu C, Herberthson M, Knutsson H, Westin C-F (2018). Influence of the size and curvedness of neural projections on the orientationally averaged diffusion MR signal. Front. Phys..

[12] Herberthson M, Yolcu C, Knutsson H, Westin C-F, Özarslan E (2019). Orientationally-averaged diffusion-attenuated magnetic resonance signal for locally-anisotropic diffusion. Sci. Rep..

[13] Afzali M, Aja-Fernández S, Jones DK (2020). Direction-averaged diffusion-weighted MRI signal using different axisymmetric B-tensor encoding schemes. Magn. Reson. Med..

[14] Yolcu, C., Herberthson, M., Westin, C.-F. & Özarslan, E. Magnetic resonance assessment of effective confinement anisotropy with orientationally-averaged single and double diffusion encoding. In *Anisotropy Across Fields and Scales* 203–223 (Springer Nature, 2021).

[15] Cheng H, Newman S, Afzali M, Fadnavis SS, Garyfallidis E (2020). Segmentation of the brain using direction-averaged signal of DWI images. Magn. Reson. Imaging.

[16] Afzali M (2020). Improving neural soma imaging using the power spectrum of the free gradient waveforms. Proc. Int. Soc. Magn. Reson. Med..

[17] Jones DK, Horsfield MA, Simmons A (1999). Optimal strategies for measuring diffusion in anisotropic systems by magnetic resonance imaging. Magn. Reson. Med..

[18] Jones D (2003). When is a DT-MRI sampling scheme truly isotropic?. Proc. Int. Soc. Magn. Reson. Med..

[19] Jones DK (2010). Diffusion MRI.

[20] Koay CG, Özarslan E, Johnson KM, Meyerand ME (2012). Sparse and optimal acquisition design for diffusion MRI and beyond. Med. Phys..

[21] Knutsson, H. Towards optimal sampling in diffusion MRI. In *International Conference on Medical Image Computing and Computer-Assisted Intervention* 3–18 (Springer, 2019).

[22] Tampu IE, Yolcu C, Knutsson H, Koay CG, Özarslan E (2019). Estimation of the Orientationally-Averaged Magnetic Resonance (MR) Signal for Characterizing Neurite Morphology.

[23] Koay CG, Özarslan E, Basser PJ (2009). A signal transformational framework for breaking the noise floor and its applications in MRI. J. Magn. Reson..

[24] Stephens MA (1974). EDF statistics for goodness of fit and some comparisons. J. Am. Stat. Assoc..

[25] Veraart J, Fieremans E, Novikov DS (2019). On the scaling behavior of water diffusion in human brain white matter. NeuroImage.

[26] McNab JA (2013). The human connectome project and beyond: Initial applications of 300 mt/m gradients. NeuroImage.

[27] Jones DK (2018). Microstructural imaging of the human brain with a ‘super-scanner’: 10 key advantages of ultra-strong gradients for diffusion MRI. NeuroImage.

[28] Rudrapatna U, Parker GD, Jamie R, Jones DK (2020). A comparative study of gradient nonlinearity correction strategies for processing diffusion data obtained with ultra-strong gradient MRI scanners. Magn. Reson. Med..

[29] Palombo M (2020). SANDI: A compartment-based model for non-invasive apparent soma and neurite imaging by diffusion MRI. NeuroImage.

[30] Dela Haije T, Özarslan E, Feragen A (2020). Enforcing necessary non-negativity constraints for common diffusion MRI models using sum of squares programming. NeuroImage.

[31] Lebedev, V. I. & Laikov, D. A quadrature formula for the sphere of the 131st algebraic order of accuracy. In *Doklady Mathematics*, Vol. 59, 477–481 (Pleiades Publishing, Ltd., 1999).

[32] Sobolev, S. L. Cubature formulas on the sphere invariant under finite groups of rotations. In *Selected Works of SL Sobolev* 461–466 (Springer, 2006).

[33] Lebedev VI (1976). Quadratures on a sphere. USSR Comput. Math. Math. Phys..

[34] Lebedev VI (1977). Spherical quadrature formulas exact to orders 25–29. Sib. Math. J..

[35] Lebedev V (1975). Values of the nodes and weights of ninth to seventeenth order Gauss–Markov quadrature formulae invariant under the octahedron group with inversion. USSR Comput. Math. Math. Phys..

[36] Beentjes, C. H. Quadrature on a spherical surface. Working note available on the website https://people.maths.ox.ac.uk/beentjes/Essays (2015).

[37] Kaasalainen M, Lu X, Vänttinen A-V (2012). Optimal computation of brightness integrals parametrized on the unit sphere. Astron. Astrophys..

[38] Descoteaux M, Angelino E, Fitzgibbons S, Deriche R (2007). Regularized, fast, and robust analytical q-ball imaging. Magn. Reson. Med..

[39] Williams EG (1999). Fourier Acoustics: Sound Radiation and Nearfield Acoustical Holography.

[40] Özarslan E, Mareci TH (2003). Generalized diffusion tensor imaging and analytical relationships between diffusion tensor imaging and high angular resolution diffusion imaging. Magn. Reson. Med..

[41] Özarslan E, Vemuri BC, Mareci TH (2005). Generalized scalar measures for diffusion MRI using trace, variance, and entropy. Magn. Reson. Med..

[42] Knutsson, H., Andersson, M. & Wiklund, J. Advanced filter design. In *Proc. SCIA* (1999).

[43] Szczepankiewicz F, Westin C-F, Knutsson H (2017). A measurement weighting scheme for optimal powder average estimation. Proc. Int. Soc. Magn. Reson. Med..

[44] Özarslan E (2013). Mean apparent propagator (MAP) MRI: A novel diffusion imaging method for mapping tissue microstructure. NeuroImage.

[45] https://vimeo.com/405277787.

[46] https://my.vanderbilt.edu/memento/sample-page/.

[47] De Luca, A. *et al.* On the generalizability of diffusion MRI signal representations across acquisition parameters, sequences and tissue types: Chronicles of the MEMENTO challenge. *bioRxiv* (2021). 10.1016/j.neuroimage.2021.118367 PMC7615259 34237442

[48] Ning L (2015). Sparse reconstruction challenge for diffusion MRI: Validation on a physical phantom to determine which acquisition scheme and analysis method to use?. Med. Image Anal..

[49] Özarslan E, Koay CG, Shepherd TM, Blackband SJ, Basser PJ (2009). Simple harmonic oscillator based reconstruction and estimation for three-dimensional q-space MRI. Proc. Int. Soc. Magn. Reson. Med..

[50] Özarslan E, Koay CG, Basser PJ (2008). Simple harmonic oscillator based estimation and reconstruction for one-dimensional q-space MR. Proc. Int. Soc. Magn. Reson. Med..

[51] Fick RH, Wassermann D, Caruyer E, Deriche R (2016). MAPL: Tissue microstructure estimation using Laplacian-regularized MAP-MRI and its application to HCP data. NeuroImage.

[52] Özarslan E, Shepherd TM, Koay CG, Blackband SJ, Basser PJ (2012). Temporal scaling characteristics of diffusion as a new MRI contrast: Findings in rat hippocampus. NeuroImage.

[53] Yablonskiy DA (2002). Quantitative in vivo assessment of lung microstructure at the alveolar level with hyperpolarized ^3^He diffusion MRI. Proc. Natl. Acad. Sci. U. S. A..

[54] Kellner E, Dhital B, Kiselev VG, Reisert M (2016). Gibbs-ringing artifact removal based on local subvoxel-shifts. Magn. Reson. Med..

[55] Andersson JL, Sotiropoulos SN (2016). An integrated approach to correction for off-resonance effects and subject movement in diffusion MR imaging. NeuroImage.

