# Supplementary material for: Computing the orientational-average of diffusion-weighted MRI signals: a comparison of different techniques
Source: Sci Rep. 2021 Jul 12;11:14345. doi: 10.1038/s41598-021-93558-1 (PMC8275746; doi:10.1038/s41598-021-93558-1)
Supplement: Supplementary file 1 — Supplementary Information 1. [file 41598_2021_93558_MOESM1_ESM.pdf]

Computing the Orientational-Average of Diffusion-Weighted MRI Signals: A Comparison of Different Techniques (Supplementary information)

Maryam Afzali[1,2,*], Hans Knutsson[3,4], Evren Özarslan[3,4,+], and Derek K Jones[1,+]

[1]Cardiff University Brain Research Imaging Centre (CUBRIC), School of Psychology, Cardiff University, Cardiff, United Kingdom
[2]Leeds Institute of Cardiovascular and Metabolic Medicine, University of Leeds, Leeds, United Kingdom
[3]Department of Biomedical Engineering, Linköping University, Linköping, SE-581 83, Sweden
[4]Center for Medical Image Science and Visualization, Linköping University, Linköping, SE-581 83, Sweden
[*]Corresponding author, Email address: AfzaliDeliganiM@cardiff.ac.uk
[+]These authors share last authorship

1 Schematic representation of point sets

Fig S1 shows a schematic representation of the shelled and non-shelled point sets used in this study (Table 1, main text).

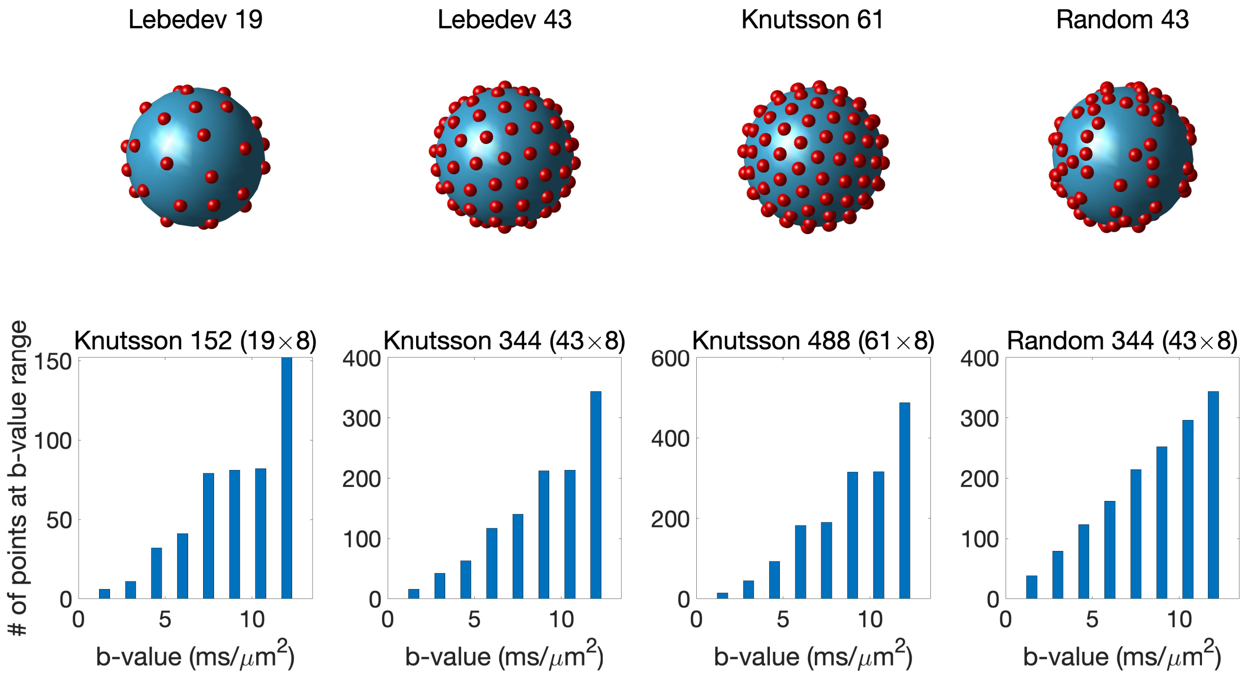

Figure S1: Shelled and non-shelled point sets. Top row shows the shelled point sets for Lebedev 19, Lebedev 43, and Knutsson 61 and random 43. Bottom row shows the cumulative number of data points acquired with a b-value less than the one represented in the x-axis as bar plots for the non-shelled point sets of Knutsson 152 (19×8), 344 (43×8), and 488 (61×8), and 334 random diffusion encoding directions.

2 Effect of noise and number of samples

Figure S2 illustrates the results obtained from 43×8 [1] samples for shelled point sets in the presence of Gaussian noise. It shows the mean and standard deviation of the estimated signal versus b-value using the MAP-MRI method [2] with $N_{\max} = 6$ for five different noise standard deviations, $\sigma_g = 0.1414, 0.0707, 0.0283, 0.0071, 0.0014$, and three different dispersions, $\kappa = 1, 9, \infty$.

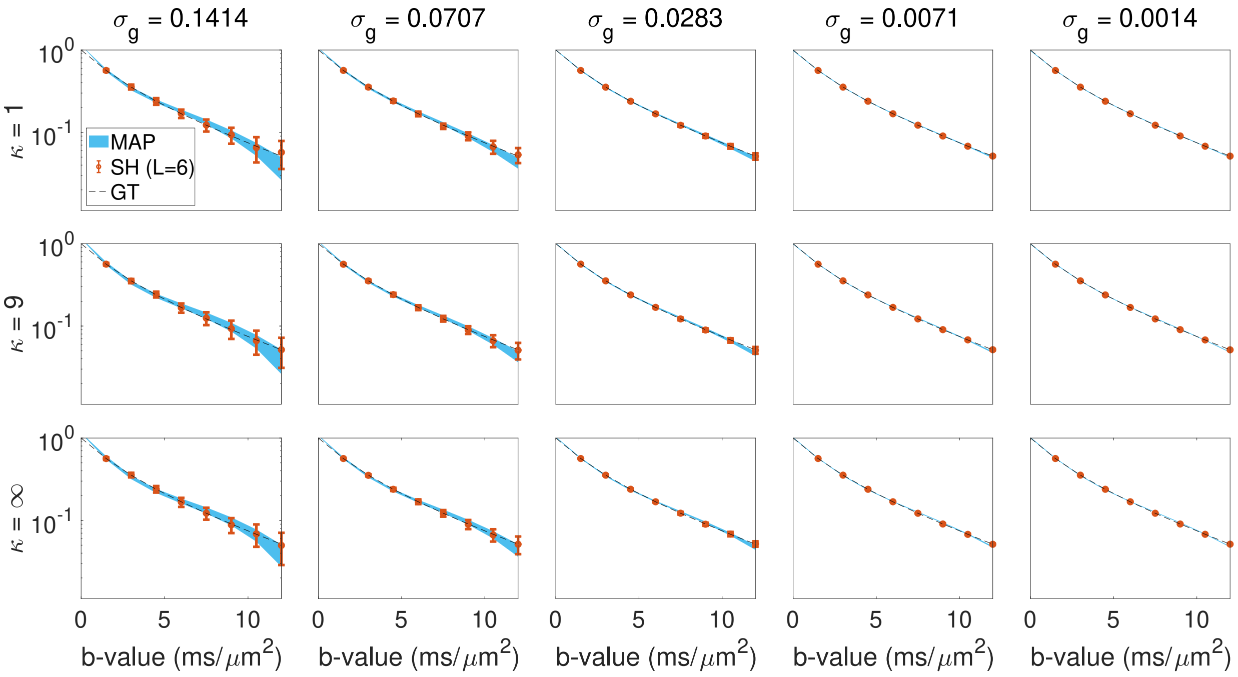

Figure S2: The results of 344 samples for shelled (43×8) [1] point sets in the presence of Gaussian noise. The mean and std of the estimated signal versus b-value using MAP-MRI method with $N_{\max} = 6$ for five different noise levels, σ_g, and three different dispersion values, κ, are illustrated. The thickness of the blue band is twice the standard deviation of the signal estimates and its center is the mean. The dashed black line shows the ground truth and the red dots and bars show the results of the SH (L = 6), spherical harmonic representation (the y-axis is scaled logarithmically).

3 Using MAP-MRI for interpolating the orientationally-averaged signal

In recently published works that employ the orientationally-averaged signal, [3, 4, 5, 6], the acquisition is performed using shell-based sampling schemes and usually restricted by acquisition time. As a result, the number of shells is not much higher than the number of parameters in the model, which could result in inaccurate parameter estimates. The ability of MAP-MRI to estimate the signal values across the q-space can be used to stabilize the fit to some solution, doing so will not necessarily improve the accuracy of the estimates and depends on MAP-MRI's ability to interpolate the signals. To illustrate this, a set of coefficients is estimated from the measurements that can

be used to provide the signal values for intermediate b-values that are not sampled. This can be especially useful when the orientationally-averaged signal is applied in multi-compartment models. To investigate the ability of MAP-MRI in interpolating the samples we used both shelled (43×8) and non-shelled 344 point sets. To generate the 'MAP, Knutsson, s8' and 'MAP, Knutsson, s43', we utilized the shelled point sets. First, the signal was orientationally-averaged using the Knutsson method [7] at $b = 1.5,\, 3,\, 4.5,\, \ldots,\, 12\, ms/\mu m^2$, then the averaged signal was used to estimate the MAP-MRI coefficients in equation (19), i.e., we used the orientationally-averaged signal using Knutsson's method as $\bar{E}$ to estimate $\kappa_{(1+N/2)00}$ in equation (19). Estimated coefficients ($\kappa_{(1+N/2)00}$) are utilized to reconstruct the signal at $b = 1.5,\, 3,\, 4.5,\, \ldots,\, 12\, ms/\mu m^2$ ('MAP, Knutsson, s8') and $b = 1.5,\, 1.75,\, 2,\, \ldots,\, 12\, ms/\mu m^2$ ('MAP, Knutsson, s43'). Note that 's8' and 's43' refer to the shelled point sets with 8, and 43 b-values, respectively, and number '43' in 's43' is independent from the number of gradient directions in 43×8 point set.
To generate 'MAP, s8' and 'MAP, s43' the shelled point set was used similar to the previous scenario, but with the distinction that we do not average the signal; all points are utilized to generate the MAP-MRI coefficients in equation (17) (κ_{jlm}). Estimated coefficients (κ_{jlm}) are utilized to reconstruct the signal at $b = 1.5,\, 3,\, 4.5,\, \ldots,\, 12\, ms/\mu m^2$ ('MAP, s8') and $b = 1.5,\, 1.75,\, 2,\, \ldots,\, 12\, ms/\mu m^2$ ('MAP, s43'). The same scheme is used to generate the results of 'MAP, ns8' and 'MAP, ns43' for the non-shelled set of 344 samples. The $N_{\max}$ used in all the experiments of this section (MAP-based interpolation) was taken to be 6.
Figure S3 illustrates the results of MAP-based interpolation. The mean and std of the d_1 and d_2 measures for different methods ('MAP, Knutsson, s8', 'MAP, Knutsson, s43', 'MAP, s8', 'MAP, s43', 'MAP, ns8', and 'MAP, ns43') are illustrated in Figure S3. Note that when the MAP-MRI coefficients were used to estimate the powder-averaged signal of $b = 1.5,\, 3,\, 4.5,\, \ldots,\, 12\, ms/\mu m^2$ the results were the same as those reported in Fig. 2 (a). Using MAP-MRI for interpolation ($b = 1.5,\, 1.75,\, 2,\, \ldots,\, 12\, ms/\mu m^2$) provided similar d_1 and d_2 compared to $b = 1.5,\, 3,\, 4.5,\, \ldots,\, 12\, ms/\mu m^2$.

4 Orientation averaging in multi-compartment models

A three-compartment model, Stick + Ball + Sphere, was used to investigate the effect of different orientation averaging techniques. The details of the model are provided in [4, 9]. Figure S4 shows the results of multi-compartment fit on in-vivo data where f_{stick}, f_{sphere}, and f_{ball} are the signal fractions of stick, sphere, and ball compartments respectively. $D_{in}^{||}$ is the parallel diffusivity inside stick, D_{ball} is the ball diffusivity, and R is the sphere radius. As the results show different methods yield different estimates for the parameters of the multi-compartment model where the results of shell-based techniques are self consistent and the results of MAP-based methods are slightly different. A likely reason is that in the MAP estimates, we enforce constraints that make the average propagator nonnegative. It is also to be noted that when $N_{max} = 6$, the regularization associated with MAP-MRI is quite strong. This may prevent overfitting in very noisy situations while suppressing some relevant features in others. These findings are consistent with those reported in Fig. 7 in the main text.

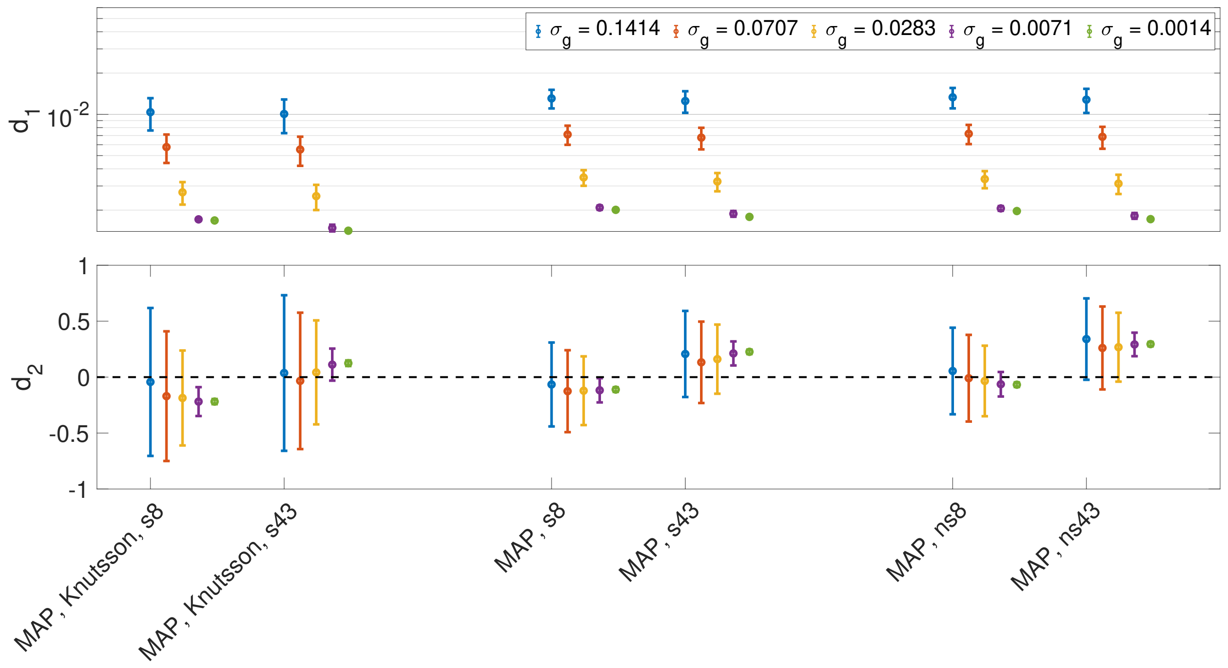

Figure S3: The results of MAP-based interpolation of the orientationally-averaged data from Knutsson method on 43×8 shelled Lebedev [1] and non-shelled point sets [8]. The mean and std of the d_1 and d_2 measures for different scenarios (the y-axis in d_1 is scaled logarithmically). Note that the $N_{\max}$ used in all the experiments in this figure is equal to 6.

References

[1] Lebedev Vyacheslav Ivanovich, Laikov DN. A quadrature formula for the sphere of the 131st algebraic order of accuracy in *Doklady Mathematics*;59:477–481Pleiades Publishing, Ltd.) 1999.

[2] Özarslan Evren, Koay Cheng Guan, Shepherd Timothy M, et al. Mean apparent propagator (MAP) MRI: a novel diffusion imaging method for mapping tissue microstructure *NeuroImage.* 2013;78:16–32.

[3] McKinnon Emilie T, Jensen Jens H, Glenn G Russell, Helpern Joseph A. Dependence on b-value of the direction-averaged diffusion-weighted imaging signal in brain *Magnetic resonance imaging.* 2017;36:121–127.

[4] Palombo Marco, Ianus Andrada, Guerreri Michele, et al. SANDI: a compartment-based model for non-invasive apparent soma and neurite imaging by diffusion MRI *NeuroImage.* 2020:116835.

[5] Afzali Maryam, Aja-Fernández Santiago, Jones Derek K. Direction-averaged diffusion-weighted MRI signal using different axisymmetric B-tensor encoding schemes *Magnetic Resonance in Medicine.* 2020.

[6] Afzali Maryam, Palombo Marco, Mueller Lars, et al. Improving neural soma imaging using the power spectrum of the free gradient waveforms in *Proc. Intl. Soc. Mag. Reson. Med.* 2020.

[7] Knutsson Hans, Andersson Mats, Wiklund Johan. Advanced filter design in *Proc SCIA* 1999.

[8] Knutsson Hans. Towards Optimal Sampling in Diffusion MRI in *International Conference on Medical Image Computing and Computer-Assisted Intervention*:3–18Springer 2018.

[9] Afzali Maryam, Nilsson Markus, Palombo Marco, Jones Derek K. SPHERIOUSLY? The challenges of estimating sphere radius non-invasively in the human brain from diffusion MRI *NeuroImage.* 2021;237:118183.

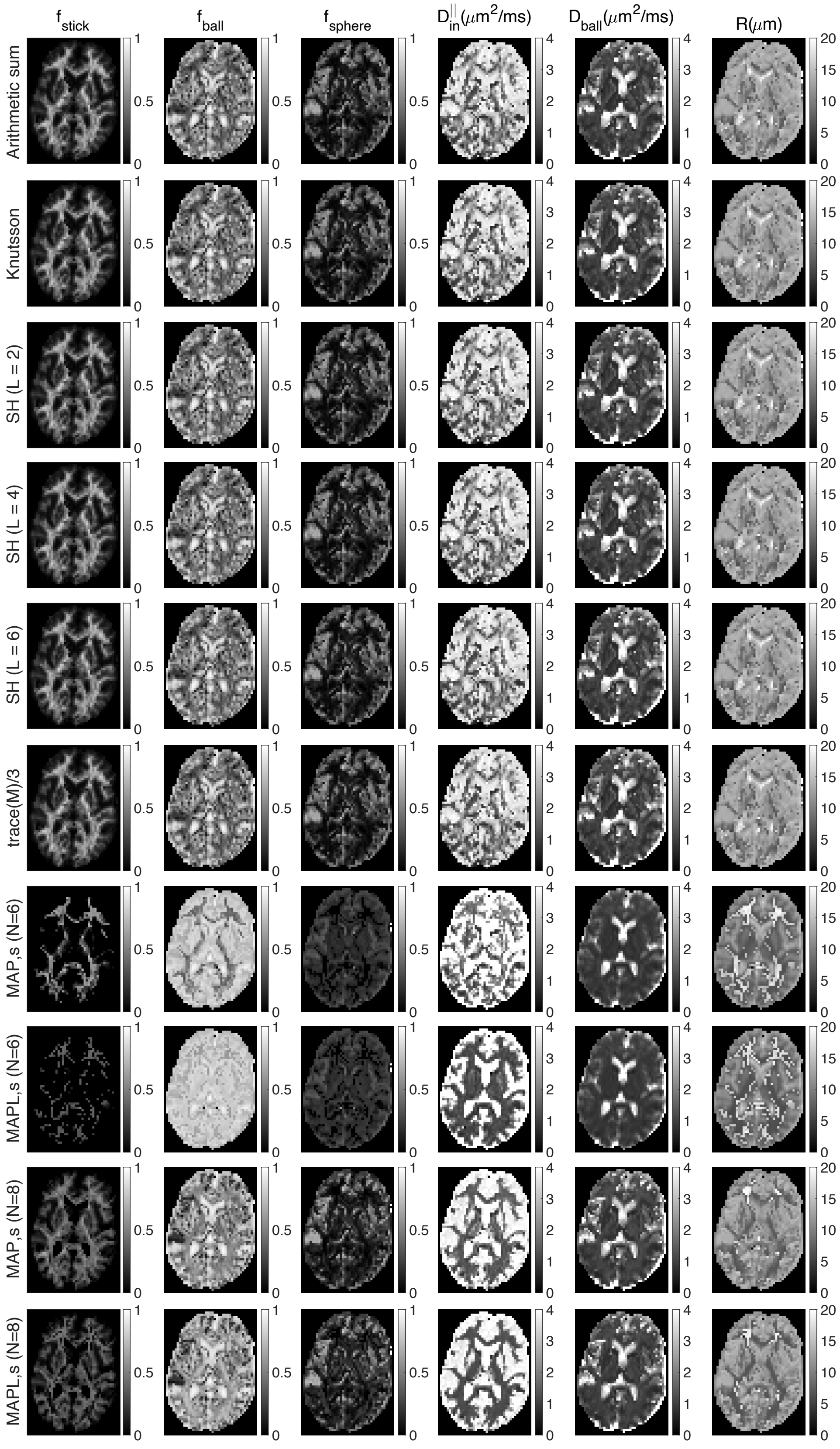

Figure S4: The results of fitting a three-compartment model to the orientationally averaged signal of in vivo data.
